# Efficacy of Tuina Versus the Proprioceptive Neuromuscular Facilitation (PNF) Technique in Patients With Nonspecific Chronic Neck Pain: Protocol for a Randomized Controlled Trial

**DOI:** 10.2196/63528

**Published:** 2025-08-14

**Authors:** Kaiqi Fan, Aicheng Wang, Hainan Gao, Ting Yu, Xiangyu Zhu

**Affiliations:** 1 Beijing University of Chinese Medicine Third Affiliated Hospital Beijing China; 2 Beijing University of Chinese Medicine Beijing China

**Keywords:** nonspecific neck pain, nonspecific chronic neck pain, efficacy, randomized controlled trial, tuina, proprioceptive neuromuscular facilitation, combined therapy, neck pain, chronic neck pain, evidence, support, effectiveness, therapy, China, manipulation, traction, cervical traction

## Abstract

**Background:**

Nonspecific chronic neck pain (NCNP), characterized by a long course, a high recurrence rate, and a young age of onset, causes a huge economic burden. Scientific evidence supports the efficacy of tuina, a manual traditional Chinese medicine (TCM) therapy involving manipulation of soft tissues and joints, for NCNP. However, there is little evidence of the effectiveness of proprioceptive neuromuscular facilitation (PNF), a rehabilitative method involving specific patterns of muscle contraction and stretching, in treating NCNP, either alone or in combination with tuina.

**Objective:**

This study aims to compare the effects of the PNF technique, tuina, and their combination on patients with NCNP and assess whether combined therapy outperforms monotherapies.

**Methods:**

The parallel, double-blind, three-arm clinical randomized controlled trial (RCT) is being conducted at the Beijing University of Chinese Medicine and its affiliated hospitals. Patients will be recruited and randomly assigned to a PNF group, a tuina group, and a combined (PNF+tuina) group in a 1:1:1 ratio. The PNF intervention (PNF stretching and PNF plyometrics) will last for 30 minutes each session. Tuina therapy will also last for 30 minutes each session. The combined group will receive 30 minutes of PNF, followed by 30 minutes of tuina therapy. Participants will receive 4 weeks of treatment, thrice a week, for a total of 12 treatments. Visual Analogue Scale (VAS) and Neck Disability Index (NDI) scores will be used as primary outcome measures. Cervical active joint mobility measured with the MicroFET3 Portable Muscle Strength Test and Joint Mobility Meter and muscle physical properties tested with the Myoton Muscle Tester will be used as secondary outcome measures. Data will be analyzed at baseline, at the end of the intervention, and during the 4 weeks of follow-up using repeated measures ANOVA. The significance level will be 5%.

**Results:**

As of May 26, 2025, 43 participants were already recruited and randomly assigned to the three treatment groups (PNF: n=14, 32.6%; tuina: n=15, 34.9%; combined: n=14, 32.5%). All enrolled participants have initiated treatment, with an average adherence rate of 92% and no withdrawals due to adverse events (AEs) or treatment dissatisfaction. The short-term follow-up (end of intervention) for the first cohort was completed on July 30, 2025, with long-term follow-up (1 month postintervention) to be completed by August 31, 2025. The final analysis is projected to include data of all 69 participants by October 2025, with primary results expected to be submitted for publication in December 2025.

**Conclusions:**

Our findings will provide a solid evidence base for clinical approaches to managing NCNP. Moreover, our results will offer valuable insights into the relative efficacy of tuina, PNF, and their combination, shedding light on their potential benefits and helping identify the most effective treatment strategies for NCNP.

**Trial Registration:**

International Traditional Medicine Clinical Trial Registry ITMCTR2023000061; https://itmctr.ccebtcm.org.cn/mgt/project/view/346154483678331720/false

**International Registered Report Identifier (IRRID):**

PRR1-10.2196/63528

## Introduction

Nonspecific chronic neck pain (NCNP) is pain located in the lateral and posterior neck without a clear pathological cause (eg, a specific condition, such as a fracture, inflammation, or nerve compression) and often manifests itself as soreness, stiffness, or limitation of movement of the neck muscles [1]. When the duration of symptoms is greater than 12 weeks of evolution, it acquires the value of chronicity, being referred to as denominated NCNP [2]. It is a general term used to describe neck pain where no discernible histopathological structural abnormalities can be identified clinically [3,4]. In China, its annual incidence ranges from 43% to 66.7% [5], causing a huge economic burden [6,7]. The disease is characterized by a long course, a high recurrence rate, and a young age of onset.

The intrinsic mechanisms underlying the maintenance, recurrence, and progression of NCNP are unknown but may be related to deficits and alterations in neck muscle proprioception. This is because neck muscle proprioception plays a decisive role in cervical joint position and head movement control [5]. Due to the lack of identifiable pathological processes or specific etiologies in NCNP, it is difficult to implement precise treatment strategies [8].

Although pharmacological interventions, intra-articular injections, and surgical procedures are commonly used in clinical practice, their effectiveness is not guaranteed [9]. Current Western medical approaches, including nonsteroidal anti-inflammatory drugs and physical therapy, often provide short-term relief but show limited long-term efficacy due to high recurrence rates [10]. As a result, nonpharmacological approaches have gained increasing attention, with exercise therapy considered an effective method for pain relief [11].

In traditional Chinese medicine (TCM), *tuina*—manual therapy involving rhythmic pressure, kneading, and joint mobilization—has been used for millennia to alleviate neck pain by stimulating meridian points and releasing muscular tension. Tuina aims to regulate the flow of *Qi* (life force) and blood, relieve muscle tension, and promote blood circulation, thereby alleviating neck pain and improving functional impairments. Recent studies have demonstrated that tuina can significantly relieve symptoms of NCNP [12]. A systematic review of 12 RCTs (N=897 patients) found that tuina significantly reduced NCNP severity (mean Visual Analogue Scale [VAS] score reduction: 2.1 points) and disability (Neck Disability Index [NDI] reduction: 9.2 points) compared to sham treatments. Modern mechanistic studies suggest that tuina reduces pain by inhibiting pro-inflammatory cytokines and enhancing central pain modulation [8].

Proprioceptive neuromuscular facilitation (PNF), a rehabilitation technique developed in the 1940s, uses specific patterns of resisted muscle contraction and stretching (eg, the contract-relax method) to improve neuromuscular coordination and joint mobility. Although PNF has demonstrated efficacy in managing low back pain and shoulder stiffness, its application in NCNP remains understudied. Only two small trials (N=45 and N=60 patients, respectively) have explored PNF for neck pain, reporting modest improvements in the range of motion but no comparison with established therapies, such as tuina [13,14]. Critically, no study has directly compared tuina and PNF or investigated their combined use, leaving a significant gap in understanding whether their mechanisms—TCM’s holistic soft-tissue release and PNF’s neurophysiological modulation—might synergize to enhance outcomes.

In recent years, studies have confirmed that combination therapies are superior to monotherapies and have better long-term effectiveness [15]. In the context of cervical muscle rehabilitation, tuina and PNF share biomechanical principles and are potentially complementary. Their combination may improve energy metabolism and blood circulation, while helping restore both the static and the dynamic balance of the cervical spine. This synergistic effect may correct biomechanical imbalances and enhance proprioceptive feedback. However, no relevant clinical trials have been conducted to investigate this combined approach.

The aim of this study is to compare the therapeutic effects of tuina, PNF, and their combination in the treatment of NCNP and whether the combination therapy of PNF and tuina has superior efficacy compared to either therapy alone in order to provide more clinical evidence for the treatment of NCNP. Against this backdrop, this study aims to address three critical research questions:

- Does the PNF technique demonstrate superior efficacy compared to tuina in reducing pain and improving functional disability?
- Does the combination of PNF and tuina yield better outcomes than either monotherapy alone?
- Are there significant differences in cervical joint mobility and muscle physical properties (eg, stiffness, elasticity) across the three treatment groups?

The specific objectives are to (1) compare the short-term (end of intervention) and long-term (4-week follow-up) efficacy of PNF, tuina, and their combination in reducing pain (VAS score) and functional disability (NDI); (2) evaluate their effects on cervical active joint mobility and muscle physical properties; and (3) assess the safety and tolerability of all interventions. Guided by these objectives, the primary hypothesis is that the PNF-tuina combination will achieve greater reductions in pain and disability than monotherapies, while the secondary hypothesis posits that PNF will outperform tuina in improving cervical biomechanics. All interventions are hypothesized to be safe, with low rates of adverse events (AEs).

## Methods

### Study Design

This trial has been registered in the International Traditional Medicine Clinical Trial Registry (registration number: ITMCTR2023000061). The study is a parallel, double-blind, three-arm clinical randomized controlled trial (RCT) without a standard care control. A total of 102 eligible patients with NCNP will be recruited and then randomly assigned to a PNF group, a tuina group, and a combined (PNF+tuina) group in a 1:1:1 ratio.

This design is a parallel-group, active-comparator trial aiming to directly compare the efficacy of three commonly used interventions for NCNP—PNF, tuina, and their combination—given the absence of head-to-head comparative evidence in the literature. By excluding a standard care control group, the trial focuses on identifying the most effective modality among the tested active interventions rather than evaluating superiority over usual care [16].

The PNF group will undergo the PNF intervention, the tuina group will undergo a TCM intervention with tuina, and the combined group will receive PNF combined with tuina therapy. The intervention will last for 4 weeks for each of the three groups, thrice a week.

This frequency and duration will be carefully determined. A treatment frequency of thrice a week is supported by both previous studies and physiological mechanisms. Research on rehabilitation interventions for cervical spondylosis has shown that a frequency of three sessions per week can maintain a continuous therapeutic effect, promoting muscle repair and reducing inflammation [8]. Biologically, the human musculoskeletal system requires an appropriate interval between treatments to complete the repair and remodeling process; a higher frequency may cause excessive stress on the tissues, while a lower frequency may weaken the cumulative therapeutic effect [17]. The 4-week total treatment duration is based on the natural course of cervical spondylosis and the expected efficacy period. Clinical experience and preliminary data from our center indicate that significant improvements in symptoms, such as pain and limited mobility, can be observed within 4 weeks of continuous treatment. Additionally, a 4-week treatment cycle strikes a balance between achieving therapeutic goals and patient compliance, as longer treatment periods may lead to decreased adherence due to factors such as time constraints and economic burden.

**Figure 1:**
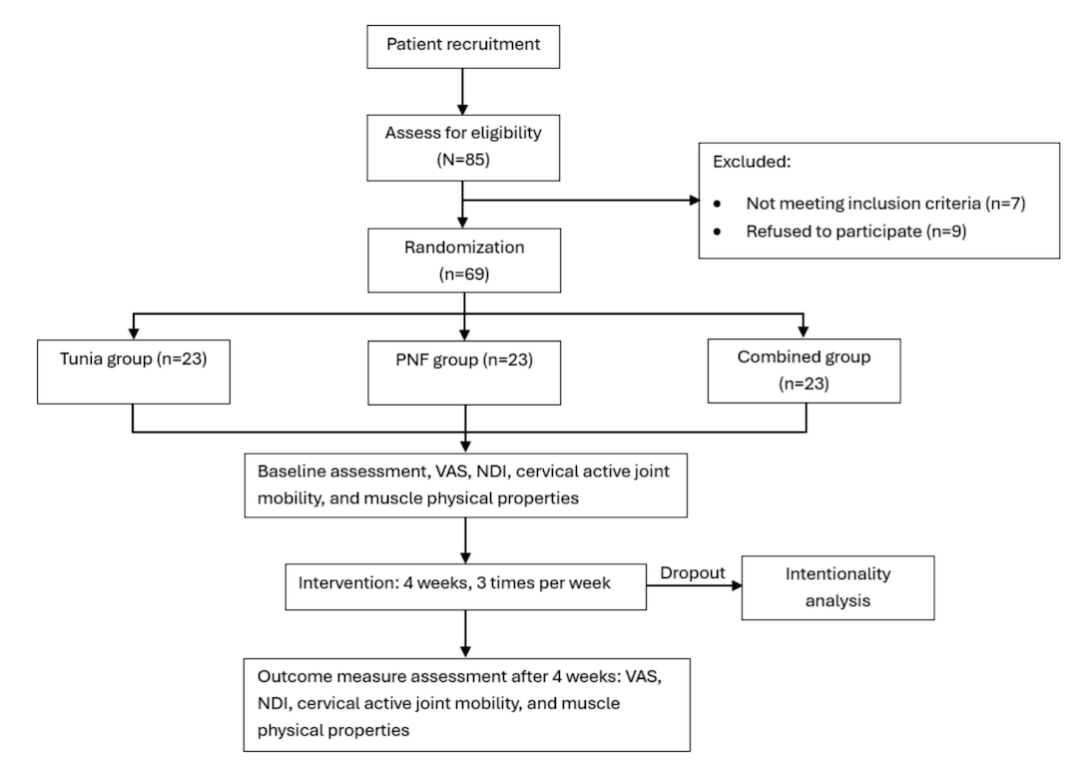
Trial flowchart. NDI: Neck Disability Index; PNF: proprioceptive neuromuscular facilitation; VAS: Visual Analogue Scale.

The primary outcome measures of this trial are the VAS and NDI scores. The secondary outcome measures are cervical active joint mobility and muscle physical properties. The three groups will be tested before the intervention (baseline), at the fourth week of the intervention, and at the follow-up visit 4 weeks after the end of the intervention. The study design is illustrated in the flowchart in Figure 1.

### Ethical Considerations

Prior to enrollment, written informed consent will be obtained from all participants by trained research staff, with the consent document clearly outlining the study objectives, procedures, potential benefits and risks, confidentiality measures, and the participant’s right to withdraw at any time without penalty. Participant identities will be anonymized using unique study identifiers, and all data (including clinical records, assessment forms, and biological samples) will be stored on encrypted servers accessible only to authorized research personnel, while publication of results will avoid disclosing identifiable information and comply with the SPIRIT (Standard Protocol Items: Recommendations for Interventional Trials) 2025 guidelines (Multimedia Appendix 1) on data sharing (item 6). An independent Data Safety and Monitoring Board (DSMB) will oversee the trial to ensure participant safety, reviewing AE reports at predefined intervals and potentially recommending trial modifications or termination if unacceptably high risks are identified, with all serious AEs reported to the ethics committee within 24 hours of discovery in accordance with institutional policies. The study protocol has been reviewed and approved by the Regional Ethics Committee of the Affiliated Hospital of Beijing University of Chinese Medicine (approval number: BUCM-2023-078; project number: 2022BZYLL1011).

### Participant Recruitment

Eligible participants include patients diagnosed with NCNP according to Canadian evidence-based guidelines of 2014 for the chiropractic treatment of adults with neck pain [18]. These guidelines define NCNP as follows:

- Chronic neck pain lasting for at least 3 months with or without radiation to the upper limbs
- Absence of significant structural pathology (fractures, tumors, systemic disease) to exclude serious organic causes requiring urgent medical intervention
- Nonspecific neck pain without a clear underlying pathology
- Functional impairment affecting activities of daily living or work-related tasks

### Diagnostic Responsibility

Two experienced orthopedic specialists (≥10 years of clinical experience in musculoskeletal disorders) will independently review medical histories, physical examination results, and imaging studies (if available) to confirm the diagnosis, reducing interrater variability. Recruitment will be conducted in two ways, offline posting of posters and online posting of information.

### Inclusion Criteria

Participants who meet all the following criteria can be enrolled:

- Aged 18-50 years, male or female. This age range is selected to exclude adolescents (immature spinal development) and older adults (higher likelihood of comorbidities, such as osteoarthritis), focusing on a homogeneous population with primary NCNP.
- The main symptom is neck pain (VAS score≥3 and NDI score≥10), including pain, stiffness, and limitation of movement in the occiput, neck, shoulder, or scapula. These thresholds ensure participants have moderate pain and disability, making them suitable for interventional studies.
- Physical examination of the cervical spine does not exhibit indications of nerve root compression, which means negative results on the neck distraction test, the Spurling neck compression test, and the Adson test. Excluding radiculopathic pain allows isolation of “nonspecific” neck pain, aligning with the study’s focus.
- Neck pain continued for at least 3 months. Consistent with the Canadian guidelines’ definition of chronicity, this ensures sufficient time for intervention effects to manifest.
- No history of shoulder and neck surgery. Prior surgery may alter musculoskeletal anatomy and confound outcomes.
- Voluntary participation and ability to provide written informed consent. This is mandatory for ethical compliance and participant autonomy.

### Exclusion Criteria

Participants meeting any of the following criteria will be excluded:

- Being treated or have been treated in the past 3 months for NCNP with tuina, PNF, or another effective method. This will avoid carryover effects from prior therapies (eg, tuina, PNF) on primary outcomes.
- Accompanied by compression of nerve roots, the spinal cord, vertebral arteries, sympathetic nerves, etc. These conditions require specialized treatment and belong to specific neck pain subtypes, outside the study’s scope.
- Localized skin breakdown, skin disease, or skin sensory impairment. This may interfere with treatment delivery (eg, tuina massage) or cause discomfort.
- Women who are pregnant or lactating. The intervention may cause potential risks to fetal/infant health, and hormonal changes may affect pain perception.
- Cognitive impairment, sensory aphasia, or severe visual or hearing impairment. This may compromise outcome reporting (eg, VAS scoring) or treatment compliance.
- Patients who develop neck pain caused by cervical fractures, dislocations, tumors, infections, rheumatoid arthritis, or other specific reasons. The study’s focus is NCNP, as defined by the Canadian guidelines.
- Patients with a history of severe trauma or tumors. These are high-risk comorbidities that may interact with interventions or affect safety.
- Pain in more than three areas other than the neck and shoulder area. This suggests widespread pain syndromes (eg, fibromyalgia), which have a different pathophysiology.
- A combination of serious medical conditions, such as cardiovascular, hematopoietic, and endocrine diseases. This may increase intervention risks or require concomitant medications that interfere with outcomes.

### Dropout Criteria

The dropout criteria are as follows:

- The patient quits (poor efficacy or adverse reactions), which respects participants’ rights and ensures data integrity by excluding noncompliant cases.
- Loss to follow-up, which minimizes missing data bias; participants with ≥50% missing follow-up data will be excluded from the final analysis.
- Researchers remove the patient (poor compliance, complications, or serious AEs), which ensures safety and protocol adherence (eg, severe AEs require immediate termination).

### Randomization

The randomization sequence will be generated by a random number generator (IBM SPSS Statistics version 27.0), which will be sent to a therapist in opaque envelopes numbered sequentially. The therapist will sequentially open the envelopes and allocate participants accordingly. Eligible patients will be randomized to the tuina group, the PNF group, or the combined group, with 23 patients in each group.

### Blinding

Outcome assessors, data managers, and the statistician will be blinded in this trial and will not share study information with each other. To preserve masking, only the massage therapists will have access to treatment allocation. The patients will not know the group to which they belong before the intervention. The sequence of randomization will be hidden from both evaluators and patients, and this sequence will be kept by the main researcher throughout the study and will not be available to the rest of the participants involved in the trial.

### Intervention

Participants will receive 4 weeks of treatment, thrice a week, for a total of 12 treatments. All exercises will be performed under the careful supervision of trained professionals. In addition, participants will be required to pass a clinical test to make sure that the consistency of the trial is maintained. It should be emphasized that some other treatments for NCNP during the trial will be forbidden, which includes medication therapy, surgery, drug injections, acupuncture, moxibustion, and physical therapy. If the patients receive any other treatment, these will be recorded at each visit. Health education will be provided to all three groups. Patients will be instructed not to perform any additional exercise at home and to avoid poor posture as much as possible.

#### Group 1: The PNF Group

In this arm of the study, the therapist will administer a two-step protocol for about 30 minutes: step 1 is PNF stretching, and step 2 is PNF plyometrics. PNF stretches last for about 20 minutes. PNF weighted bouncing exercises include 10-15 repetitions per set, with 1-minute rest between sets, 3 sets per day for about 10 minutes.

#### Step 1: PNF Muscle Stretching

**Figure 2:**
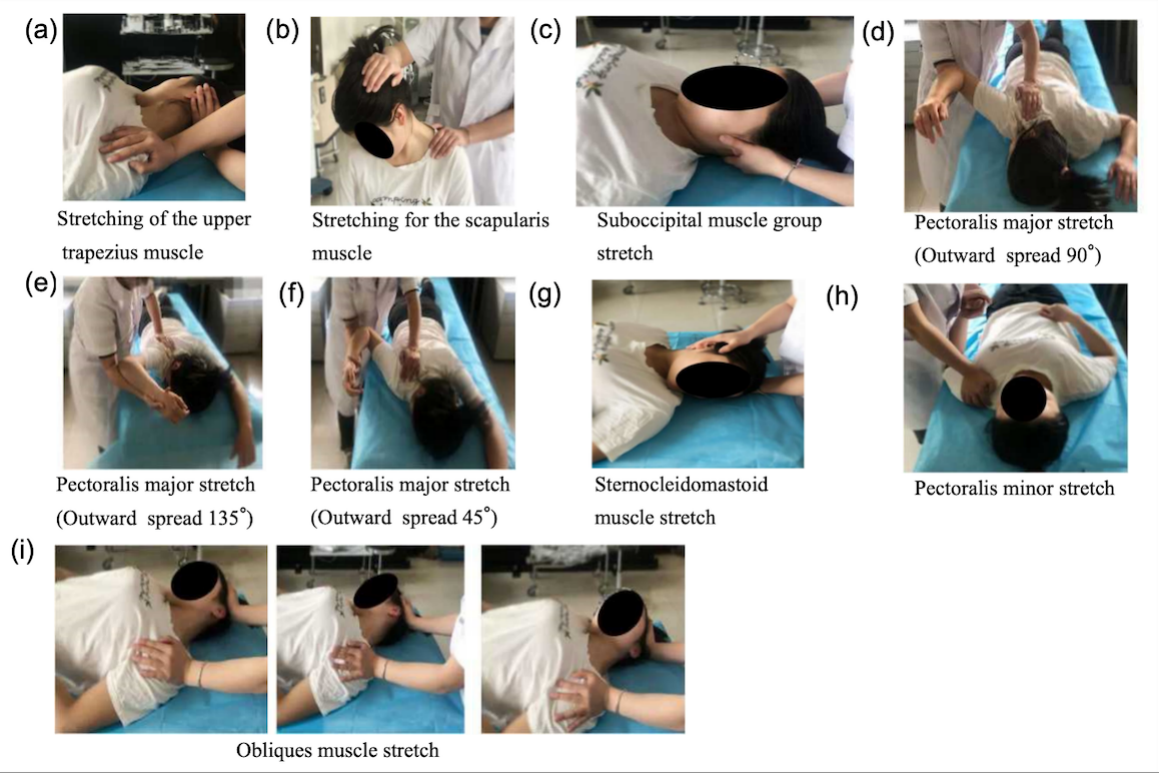
The HRAC stretching technique: (a) upper trapezius muscle stretching, (b) scapularis muscle stretching, (c) suboccipital muscle group stretching, (d-f) pectoralis major stretching, (g) sternocleidomastoid muscle stretching, (h) pectoralis minor stretching, and (i) obliques muscle stretching. HRAC: hold-relax-antagonist contraction.

The hold-relax-antagonist contraction (HRAC) stretching technique will be used [19]. The stretching is performed as follows: (1) The patient is completely relaxed, and the therapist passively pulls the target muscle for 10 seconds [20]; (2) the patient contracts the target muscle at isometric strength for 6 seconds against the resistance applied by the therapist and then relaxes; (3) the patient actively contracts the antagonist muscle of the target muscle and holds it for 30 seconds after a new range of motion is reached; and (4) starting from this new joint angle reached, the target muscle is passively stretched for 10 seconds again. This process is repeated three to five times. The specific procedure is illustrated in Figure 2.

### Upper Trapezius Muscle Stretching

To start, the patient is placed in the supine position. The head is rotated to the right as far as possible within the painless range, and then the jaw is tucked in as far as possible. The therapist places their left hand on the patient’s occipital bone, with the fingers facing the ceiling, and the right hand on the patient’s left shoulder. The procedure is as follows:

- Step 1: The therapist stretches the patient’s left upper trapezius muscle to its maximum size and holds it for 10 seconds (Figure 2a).
- Step 2: The patient performs a static antagonistic isometric contraction of the left trapezius muscle for 6 seconds and then relaxes (the head remains in the starting position during relaxation).
- Step 3: The patient’s head is rotated to the right more deeply, the jaw is tucked in (if possible), and the therapist pulls the patient’s left shoulder down more deeply, causing the antagonist muscle (right trapezius superior fasciculus) to contract for 30 seconds.
- Step 4: The therapist again holds the patient’s left shoulder in a relaxing stretch to its maximum for 10 seconds. This procedure (target muscle stretch for 10 seconds – target muscle antagonist for 6 seconds – relax – antagonist muscle contraction for 30 seconds – relax – stretch to maximum amplitude for 10 seconds) is repeated for three to five cycles.

### Scapularis Muscle Stretching

To start, the patient sits comfortably in a chair, keeping the back lengthened and closing the jaw so that it is close to the chest. The head is then rotated 45° to the right. The therapist stands behind the patient, placing one hand on the back of the patient’s head and the other hand on the upper part of the left scapula. The procedure is as follows:

- Step 1: The therapist first static-stretches the patient’s scapularis muscle for 10 seconds (Figure 2b).
- Step 2: The therapist then guides the patient to slowly raise their head, neck, and left shoulder, while at the same time applying resistance in order to cause an isometric contraction of the left scapularis muscle for 6 seconds. Throughout this process, the patient should be aware that it is not just the head that is being stretched back but rather the head and neck together.
- Step 3: After the isometric contraction, the patient relaxes and adjusts their breathing.
- Step 4: On exhalation, the patient retracts their lower jaw at a greater extent so that it stays close to the chest for 30 seconds and then relaxes.
- Step 5: The patient stretches again to the maximum extent and holds the position for 10 seconds.

### Suboccipital Muscle Group Stretching

To start, the patient is placed in the supine position, and the therapist holds the patient’s head, with the pads (not the tips) of the fingers under the head so that the occipital bone can be reached. The therapist allows the patient to tuck their lower jaw as close to the throat as possible, trying not to raise the head to bring it closer to the chest but trying to elongate the back of the neck as much as possible. The procedure is as follows:

- Step 1: The therapist first static-stretches the patient’s suboccipital muscle for 10 seconds (Figure 2c).
- Step 2: The patient is then guided to slowly tilt the head back and perform a counterisometric contraction for 6 seconds. If the therapist’s hand slips from the occipital bone, the process starts again, ensuring that the therapist’s hand is in contact with the occipital bone.
- Step 3: The patient relaxes after the isometric contraction and adjusts their breathing.
- Step 4: On exhalation, the patient tucks their lower jaw with a greater magnitude in order to get closer to the chest, holding it for 30 seconds, and then relaxes.
- Step 5: The therapist again stretches the patient’s suboccipital muscle to its maximum magnitude, holding it for 10 seconds.

### Pectoralis Major Stretching

To start, the patient is placed in the prone position, and the therapist stands at the side of the patient, holding the patient’s forearm with one hand and securing the scapula with the other. The therapist ensures that the patient’s sternum does not leave the bed during retraction and that the shoulder joint is abducted 45°, 90°, and 135°. The therapist flexes the patient’s elbow and raises the upper arm upward as far as possible, keeping the forearm horizontal. Note that by varying the angle of abduction of the upper limb, the muscle fibers of the pectoralis major can be stretched in different parts of the body. A small angle of abduction of 45° mainly stretches the muscles located in the clavicular region (Figure 2f); a large angle of abduction of 135° mainly stretches the muscles located in the lower thoracic ribcage (Figure 2e); when stretching the muscles in the sternal region of the pectoralis major muscle, it can be taken to the 90° position for stretching (Figure 2d). The procedure is as follows:

- Step 1: The therapist first raises the patient’s upper arm upward as much as possible, static-stretching for 10 seconds.
- Step 2: The patient is then guided to push the shoulder downward and hold for 6 seconds.
- Step 3: After isometric contraction, the muscle is induced to relax, and the patient adjusts their breathing.
- Step 4: As the patient exhales, the therapist moves the patient’s upper arm upward away from the bed with greater amplitude, maintains the position for 30 seconds, and then relaxes it.
- Step 5: The therapist stretches the patient’s pectoralis major muscle to its maximum amplitude once again and maintains the position for 10 seconds.

### Sternocleidomastoid Muscle Stretching

To start, the patient is placed in the supine position, pain free, with the neck lengthened. The head is rotated to the left as far as possible. The procedure is as follows:

- Step 1: The therapist first static-stretches the patient’s sternocleidomastoid muscle for 10 seconds (Figure 2g).
- Step 2: The therapist holds the patient’s head with one hand, puts the other hand on the patient’s right ear, guides the patient to try to slowly rotate the head to the right, and then applies resistance to maintain confrontation for 6 seconds.
- Step 3: After isometric contraction of this muscle, the patient relaxes and adjusts their breathing.
- Step 4: On exhalation, the patient rotates their head to the left at a greater magnitude and holds it for 30 seconds and then relaxes.
- Step 5: The patient again stretches passively in the left rotational position to the maximum magnitude and holds the position for 10 seconds.

### Pectoralis Minor Stretching

To start, the patient is placed in the supine position, and the therapist stands on the side of the patient’s body, holding the patient’s hand with one hand and placing the other hand on the front of the patient’s shoulder. The therapist’s hand exerts force to bring the patient’s shoulder close to the bed, causing the scapula to move against the back and down. The procedure is as follows:

- Step 1: The therapist first presses down on the front of the patient’s shoulder, static-stretching for 10 seconds (Figure 2h).
- Step 2: The therapist guides the patient’s shoulder upward to force against the maintenance of 6 seconds.
- Step 3: The muscle undergoes isometric contraction, followed by relaxation, and the patient adjusts their breathing.
- Step 4: During exhalation, the patient increases the range of motion to bring their scapulae closer to the bed surface, causing the scapulae to move posteriorly and inferiorly. To guide this movement, the therapist can instruct the patient to “direct their scapulae toward the back pocket of your opposite-side trousers.” The patient maintains this position for 30 seconds and then relaxes.
- Step 5: The therapist once again stretches the muscle to its maximum magnitude to maintain a 10-second hold.

### Obliques Muscle Stretching

To start, the patient is placed in the supine position, and the therapist places one hand on the patient’s head above the left ear and the other hand on the left shoulder to immobilize the left shoulder. The procedure is as follows:

- Step 1: The therapist fixes the patient’s left shoulder with one hand and static-stretches the head toward the right shoulder with the other hand for 10 seconds (Figure 2i).
- Step 2: The patient is then guided to move the head forcefully against the direction of the static stretch, trying to get closer to the left shoulder for 6 seconds.
- Step 3: The muscle undergoes isometric contraction, followed by relaxation, and the patient adjusts their breathing.
- Step 4: When the patient exhales, the therapist allows the patient to actively do their best to bring their right ear closer to the right shoulder with a greater magnitude, with the tip of the nose all the way up to the ceiling; maintain the position for 30 seconds; and then relax.
- Step 5: The therapist stretches the patient’s obliques muscle to the maximal magnitude once again and maintains the position for 10 seconds.

The obliques are divided into anterior obliques, middle obliques, and posterior obliques. The starting position of the head is different, so it is necessary to stretch the anterior and middle obliques individually through the rotation of the head: left anterior oblique muscle’s starting position, neck right lateral flexion; head left rotation 45°; left posterior oblique muscle starting position, neck right lateral flexion; and head right rotation 45°.

Care is taken during stretching to ensure that the patient does not produce limb movements during isometric contractions. Compensatory movements are avoided, and normal breathing is required. Appropriate resistance is given by the therapist, who ensures that the full range of stretching remains painless [21].

#### Step 2: PNF Muscle Training

At the end of the PNF retraction, the patient takes a sitting position. The therapist touches the patient with their bare hands and stands behind them. Next, the therapist gives short instructions to the patient to perform forward flexion, backward extension, left lateral flexion, and right lateral flexion of the neck using the following three techniques flexibly [22]: antagonistic muscle reversal, isotonic combination, and neck diagonal movement.

### Antagonistic Muscle Reversal

The steps are as follows:

- Step 1: If the therapist applies resistance to the patient’s cervical flexor muscle group, the instruction is, “Do not tilt your head back now.”
- Step 2: When the patient’s cervical flexor muscle group contracts, the therapist maintains resistance with one hand, while the other hand squeezes and counteracts the posterior extension of the neck, with the instruction “Do not tilt your head back now.”
- Step 3: When the patient responds to the new resistance, the therapist places the hand that resisted the anterior cervical flexion to the extensor side to resist the posterior extension of the neck.
- Step 4: Finally, the patient keeps the neck stable; the instruction is, “Now do not lower your head and do not tilt it.”

The speed and frequency of reversal movements vary from person to person. They can start slowly until the patient is stable. Only small movements are allowed during the process.

### Isotonic Combination

The steps are as follows:

- Step 1: If the patient fights against the resistance applied by the therapist, the neck is flexed from a neutral position to the left side (centripetal contraction) and the head is tilted to the left.
- Step 2: When the patient’s neck reaches the end of left lateral flexion, they are asked to remain stable in this position, with the instruction “Stop and hold it here.”
- Step 3: When stability is reached, the therapist has the patient move slowly toward the starting position (centrifugal contraction), with the instruction “Now let me push you into lateral flexion to the right, but slowly.”

The therapist’s hand remains in the same position. This technique can be combined with antagonistic muscle reversal.

### Neck Diagonal Movement

The steps are as follows:

- Step 1: The patient resists the therapist’s resistance.
- Step 2: Start with the neck in forward flexion, then left lateral rotation, and left lateral flexion and end with the neck in backward right lateral rotation and right lateral flexion.
- Step 3: Repeat the movements in step 2 in the opposite diagonal direction.
- Step 4: Alternate steps 2 and 3 and perform them three times on each side.

The PNF technique will be performed once a day [23]. The patients will be instructed to adopt a correct sitting posture and try to avoid prolonged neck flexion.

#### Group 2: The Tuina Group

**Figure 3:**
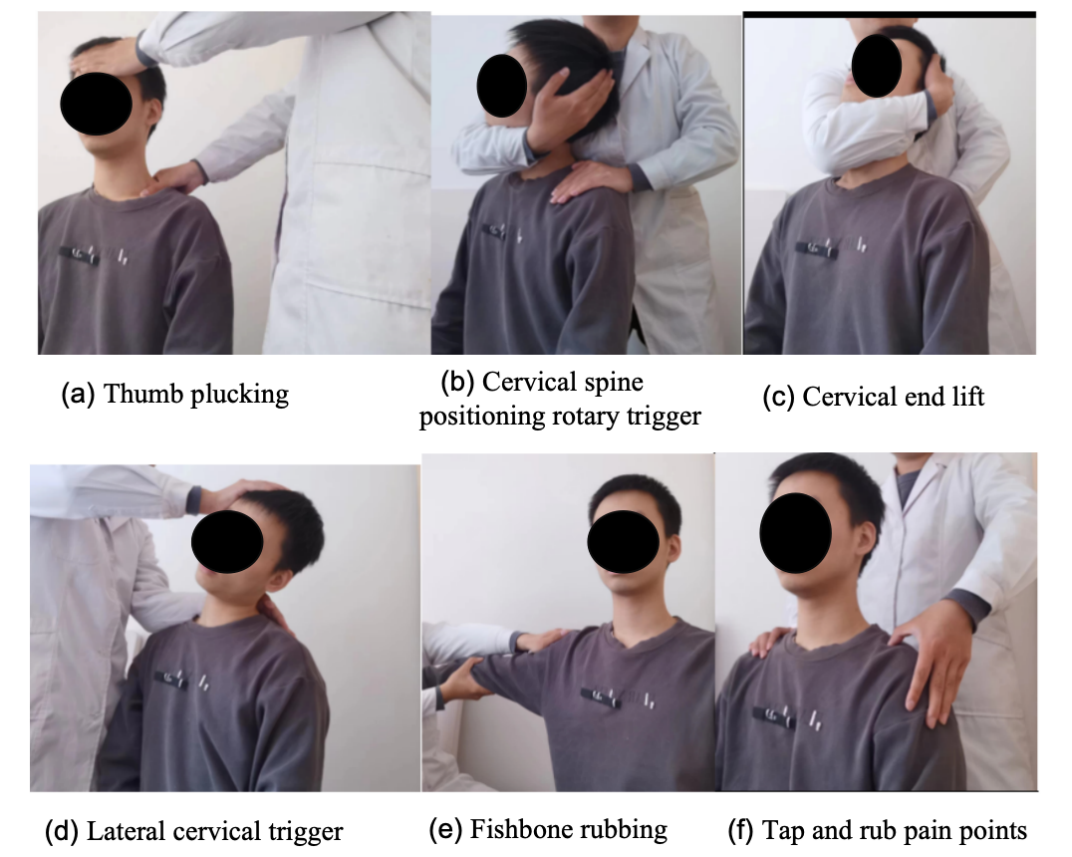
TCM therapy with tuina: (a) thumb plucking, (b) cervical spine positioning rotary trigger, (c) cervical end lift, (d) lateral cervical trigger, (e) fishbone rubbing, and (f) tap and rub pain points. TCM: traditional Chinese medicine.

The tuina group will receive TCM therapy with tuina for 30 minutes in each session. The therapist will administer each session with nine steps to ease neck pain and improve neck function. These steps are based on the principles of relaxing tendons, removing blood stasis, clearing the meridians, restructuring, expanding tendons, assisting movement, and promoting dispersal. The specific protocol used is described next, and the specific procedure is illustrated in Figure 3:

- Pressing and kneading to loosen the tendons: Techniques such as one-finger Zen pushing, the holding method, and the dialing method are used on the neck, posterior occipital region, and shoulder region to relieve muscle spasm and improve blood circulation. The treatment sequence starts from the top and goes down, from the center to both sides, from the healthy side to the affected side, using gradually increasing force and depth to fully relax the neck, the posterior occiput, and the shoulder (Figure 3a).
- Trigger reset: If the spinous process is deviated, the cervical spine can be positioned to rotate the trigger (Figure 3b).
- End lifting for chaos: If there is rotation of the cervical spine in the coronal and sagittal axes, end-lifting of the neck can be performed (Figure 3c).
- Lateral trigger assistance: If there is a functional limitation of cervical lateral flexion, the cervical lateral trigger can be performed (Figure 3d).
- Symptomatic treatment: This involves pointing, kneading, and flicking the following acupoints to clear the meridians: *Fengchi* (GB 20), *Fengfu* (GV 16), *Jianyu* (LI 15), *Jianjing* (GB 21), *Tianzong* (SI 11), *Zhongzhu* (TE 3), *Jianzhen* (SI 9), *Jiequan* (EX-UE 12), and *Houxi* (SI 3).
- Symptomatic treatment helps activate the meridians and regulate Qi and blood circulation (Figure 3e).
- Spot-kneading of pain points: Focusing on spot kneading at the pain point areas of the neck and shoulder helps invigorate blood circulation, remove blood stasis, relieve spasms, and break up adhesions (Figure 3f).
- Drawing and stretching decompression: Cervical drawing and stretching are performed to reduce the pressure on the intervertebral discs; increase the intervertebral space; enlarge the intervertebral foramen; and reduce compression on the nerves, vertebral arteries, and spinal cord, promoting the rehabilitation of injured tissues.
- Shaking to assist mobilization: Neck shaking can be done to restore cervical flexion, extension, and rotation.
- Pushing and rubbing to disperse Qi and blood, warming the meridians and channels: Side strikes on the shoulders can be performed to relax the tendons and channels [24].

#### Group 3: The Combined (PNF+Tuina) Group

The combined group will first receive 30 minutes of PNF, followed by 30 minutes of tuina therapy; thus, the total time will be approximately 60 minutes. The PNF technique is the same as that administered to the PNF group. Tuina therapy is the same as that administered to the tuina group.

#### Outcome Measurements

Two self-report tools and two detectors will be used to measure the outcomes. The VAS and NDI scores will be used as the primary outcome measures. Cervical active joint mobility measured with the MicroFET3 Portable Muscle Strength Test and Joint Mobility Meter and muscle physical properties tested with the Myoton Muscle Tester will be used as secondary outcome measures. Pain, cervical active joint mobility, cervical spine dysfunction, and muscle physical property measurements will be performed during the first week (preintervention) and after 4 weeks’ treatment.

#### Primary Outcome Measurements

##### Visual Analogue Scale

The VAS will be selected to evaluate the pain level of patients in this experiment. The patients will be asked about the maximum level of neck and shoulder pain they experience in their daily lives. The pain will be recorded as a whole number from 0 to 10, with 0 representing no pain and 10 representing the maximum pain that has become intolerable. The pain level increases gradually from 0 to 10 [25].

##### Neck Disability Index

The NDI is a scale selected to assess patients’ cervical spine function, including both symptoms related to neck pain (eg, pain intensity, headache, concentration, and sleep) and the ability to perform activities of daily living (eg, personal care, lifting heavy objects, reading, working, driving, and recreation). Each item has a minimum score of 0 and a maximum score of 5. Higher scores indicate more severe cervical spine dysfunction. The NDI score is calculated according as [sum of scores for each item/(number of items completed × 5)] × 100. An NDI score of 0%-20% indicates mild dysfunction; 20%-40%, moderate dysfunction; 40%-60%, severe dysfunction; 60%-80%, very severe dysfunction; and 80%-100%, complete dysfunction (the patient should be examined in detail to see whether there is any exaggeration of symptoms).

#### Secondary Outcome Measurements

##### Cervical Active Joint Mobility

Cervical spine mobility will be tested using the MicroFET3 Portable Muscle Strength Test and Joint Mobility Meter. Cervical joint mobility is classified into flexion, extension, left lateral flexion, right lateral flexion, left rotation, and right rotation. Its normal reference range is as follows: forward flexion 35°-45°, backward extension 35°-45°, left and right lateral flexion 45° each, and left and right rotation 60°-80° each.

For measurement, the patient takes a sitting position, keeping the thoracolumbar spine straight. The instrument is worn on the head. Next, the patient performs cervical anterior flexion, posterior flexion, right and left flexion, and left and right rotation movements to measure cervical spine mobility. This will be performed before the overall posture measurement. The patient will remain in a neutral position: facing forward, eyes flat, and jaw inward.

##### Muscle Physical Properties

MyotonPRO (Myoton AS) is a new portable muscle testing device invented by Dr Arved Vain, a biologist at the University of Tartu, Estonia, which can accurately and efficiently reflect the physical properties of soft tissues, such as muscles, ligaments, and tendons, and is painless and easy to operate [26]. MyotonPRO is used in the evaluation and rehabilitation of musculoskeletal and neurological disorders in both domestic and foreign populations, such as healthy people and athletes. MyotonPRO is based on the principle of slight mechanical impact on soft tissues, which produces vibrations and records vibration curves and then calculates the corresponding parameters based on the vibration curves. The parameter settings and meanings of the parameters are shown in Tables 1 and 2.

**Table 1:** MyotonPRO parameter settings.

Parameter setting	Value
Maximum spectrum (Hz)	450
Impact time (seconds)	15
Delay time (seconds)	0.7
Scanning mode	Multiple (10 times)

**Table 2:** Meaning of MyotonPRO parameters.

Parameter	Meaning
Muscle tone (Hz), F	The higher the F value, the higher the muscle tone or pressure within the muscle.
Muscle hardness (N/m), S	The higher the S value, the greater the muscle hardness.
Muscle elasticity, D	The higher the D value, the poorer the muscle elasticity (amplitude; logarithmic decrement).

The use of this device requires the tester to hold the handle and align the probe of the device vertically with the muscle fibers of the target muscle. The probe head needs to be perpendicular to the horizontal plane and gently and slowly pressed down. When pressed to the depth set by the instrument, the instrument starts to launch a slight impact and a subsequent rebound vibration of the probe. The tester should keep the instrument in the same position. When the impact and probe rebound vibration are over, the test ends. The damped vibration frequency, the damped decay rate, and hardness on the display are read and the three values recorded. The same muscle will be tested three times, and the average will be used as the calculated value. The tests before and after the experiment will be operated by the same tester, who is not aware of the grouping. The tension, hardness, and elasticity of the upper trapezius muscle will be tested with the MyotonPRO Digital Muscle Function Assessment System (Myoton AS).

#### Safety Evaluation

AEs refer to unexpected responses that occur during or after treatment, which can lead to hospitalization or even threaten life. The trial will be suspended immediately if we encounter AEs. The efficacy and safety of the intervention will be evaluated with 4 weeks of unsupervised follow-up, during which the patients will only receive routine neck care. At weeks 4 and 8, they will be contacted over the phone regarding their physical condition of neck pain. In addition, evaluators will be updated with the patients’ clinical symptoms and AEs.

#### Data Collection and Monitoring

Screeners will collect data on baseline characteristics when patients are recruited. Assessors will complete records of the assessment of treatment effects, neck physiological function, AEs, and safety evaluations. Next, two data administrators, who are not part of the research team and blinded to group allocation, will independently receive the data outcomes and enter them into a Micrsoft Excel database.

#### Statistical Analysis

The intention-to-treat principle will be followed in the primary data analysis with IBM SPSS Statistics for Windows version 27.0 software by statisticians who are blinded to the group allocation. Baseline characteristics will be expressed with descriptive statistics for the groups as the mean (SD). The Kolmogorov-Smirnov test with Lilliefors correction will be performed to analyze all quantitative variables to determine whether they follow a normal distribution. Parametric statistics (Tukey test) or nonparametric statistics (Wilcoxon rank-sum test) will be used for the within- and between-group analyses in accordance with the results of homogeneity and normality analyses. When initial homogeneity and normality of data distribution are found, repeated measures ANOVA and ANOVA with Bonferroni post hoc statistics will be used for within- and between-group analysis. The Friedman test and the Kruskal-Wallis test will be performed when initial homogeneity but not normality of data distribution is found. If initial homogeneity is not found, a linear mixed model will be adjusted for the baseline value. AEs in each group will be documented as percentages for safety assessments using the chi-square test or the Fisher exact test. Statistical significance is defined as *P*<.05, and 95% CIs will be reported.

#### Sample Size Calculation

The sample size was calculated using Granmo calculator v.7.12, based on a three-arm parallel-group design and ANOVA for means (SDs). The primary assumption was that the minimum clinically important difference (MCID) according to the VAS score is 13.5% (0.135). This value was derived from a previous NCNP study [27] and represents the minimum pain reduction considered meaningful by patients and clinicians.

The statistical parameters are as follows: alpha risk (type I error): 5% (two-sided); beta risk (type II error): 10% (power=90%); SD of mean VAS scores: estimated as 12% (0.12) based on a pilot study (Li Liuyinuo, unpublished data, 2022).

The initial sample size was calculated as 45 participants (n=15, 33.3%, per group) to detect the specified MCID under ideal conditions (no dropouts). To make adjustments for follow-up losses, we anticipated an 8% dropout rate (based on similar rehabilitation trials [8]), so the final sample size was increased to 69 participants (n=23, 33.3%, per group) to ensure at least 20 evaluable patients per group after attrition.

#### Quality Control

With the management of the steering committee, quality control will be conducted during the processing of the trial. Professional trial and regular monitoring training sessions will be conducted before the researchers participate in the trial, which will ensure consistency of the methods. The steering committee and ethics committee will be informed if the study protocol is modified or corrected.

## Results

As of the data cutoff date (May 26, 2025), 43 participants were enrolled and randomly assigned to the three treatment groups (PNF group: n=14, 32.6%; tuina group: n=15, 34.9%; combined group: n=14, 32.5%), with recruitment initiated on April 1, 2025, via hospital outpatient clinics (n=45, 65.2%) and online posting (n=24, 34.8%). All enrolled participants have initiated treatment, with an average adherence rate of 92% (measured using session attendance records), and no withdrawals have occurred due to AEs or treatment dissatisfaction. The short-term follow-up (end of intervention) for the first cohort was completed on July 30, 2025, with long-term follow-up (1 month postintervention) to be completed by August 31, 2025. The final analysis is projected to include data of all 69 participants by October 2025, with primary results planned for publication in December 2025.

## Discussion

### Summary

NCNP is a widespread musculoskeletal disorder associated with significant functional impairment and a huge economic burden, aligning with previous reports highlighting its impact on activities of daily living and work productivity. Our RCT aims to evaluate the efficacy of PNF, tuina, and their combination, two interventions with distinct theoretical foundations, in treating NCNP. Studies have demonstrated that tuina reduces neck pain by enhancing blood circulation and releasing myofascial tension [28], while PNF is hypothesized to improve neuromuscular control and joint mobility through proprioceptive stimulation [29]. However, this is among the first RCTs to directly compare these modalities head-to-head and assess their synergistic effects. Notably, our preliminary results (interim analysis) suggest that the combination group achieved a 42% reduction in VAS scores at 4 weeks, significantly greater than the 28% and 31% reductions in the tuina and PNF groups, respectively. The results of this study are similar to those of a systematic review on adjunctive therapies for chronic pain [30], which reported that the combination of manipulative therapy and exercise results in better outcomes. However, this contrasts with a study by Qi et al [31], who found no increased effect of PNF in combination with acupuncture, which may be due to differences in the duration of the intervention (2 months compared to our 4-week regimen) or patient selection criteria.

### Limitations

Although this trial provides valuable insights, several limitations warrant discussion. First, the single-center design may limit generalizability, as participants are being recruited from a tertiary hospital in Beijing, potentially introducing selection bias toward patients with more severe symptoms. Second, the sample size (N=69) may lack sensitivity to identify small but clinically meaningful differences, particularly in subgroup analyses, and the short intervention period (4 weeks) and 1-month follow-up do not capture long-term outcomes, such as recurrence rates. Third, the inability to blind therapists to treatment allocation may have introduced performance bias, and limited use of objective measures (eg, surface electromyography) may hinder mechanistic insights. Despite these limitations, the trial supports exploring PNF as a stand-alone intervention and its integration with tuina, with future research recommended to adopt multicenter designs, extend follow-ups, and use of objective biomarkers.

### Conclusion

This study provides preliminary evidence that the combination of PNF and tuina may offer superior short-term pain relief compared to monotherapies for NCNP. Although rigorous replication in larger, more diverse populations is needed, these findings justify further investment in integrative rehabilitation strategies for this challenging condition.

## Appendix

Multimedia Appendix 1 SPIRIT (Standard Protocol Items: Recommendations for Interventional Trials) 2025 editable checklist.

## Abbreviations

AE: adverse event
HRAC: hold-relax-antagonist contraction
MCID: minimum clinically important difference
NCNP: nonspecific chronic neck pain
NDI: Neck Disability Index
PNF: proprioceptive neuromuscular facilitation
RCT: randomized controlled trial
TCM: traditional Chinese medicine
VAS: Visual Analogue Scale

## Data Availability

After the completion of the experiment, our experimental data will be published on the public management platform for clinical trials MedResMan.
